# Accuracy of an experimental whole-blood test for detecting reactivation of echinococcal cysts

**DOI:** 10.1371/journal.pntd.0009648

**Published:** 2021-08-20

**Authors:** Linda Petrone, Francesca Tamarozzi, Ambra Vola, Maria Angeles Gomez Morales, Alessandra Ludovisi, Saeid Najafi Fard, Mara Mariconti, Enrico Brunetti, Delia Goletti

**Affiliations:** 1 Translational Research Unit, National Institute for Infectious Diseases (INMI) “Lazzaro Spallanzani”-IRCCS, Rome, Italy; 2 Department of Infectious-Tropical Diseases and Microbiology, IRCCS Sacro Cuore Don Calabria Hospital, Negrar di Valpolicella, Verona, Italy; 3 Microbiology and Virology Unit, Diagnostic Medicine Department, IRCCS San Matteo Hospital Foundation, Pavia, Italy; 4 Foodborne and Neglected Parasitic Diseases Unit, Department of Infectious Diseases, Istituto Superiore di Sanità, Rome, Italy; 5 Infectious Diseases and Immunology, IRCCS San Matteo Hospital Foundation, Pavia, Italy; 6 Department of Clinical, Surgical, Diagnostic and Pediatric Sciences, University of Pavia, Pavia, Italy; University of Utah, UNITED STATES

## Abstract

**Background:**

Cystic echinococcosis (CE) is a complex disease for which clear understanding of clinical manifestations is needed to avoid misdiagnosis, inappropriate treatment, and severe complications. We evaluated the accuracy of a whole-blood stimulation test based on Interleukin (IL)-4 detection in response to Antigen B (AgB) of *Echinococcus granulosus sensu lato* to discriminate cyst viability and detect cyst reactivation in patients with hepatic CE.

**Methodology/Principal findings:**

Thirty patients with CE3b cysts and 37 patients with spontaneously-inactivated CE4-CE5 cysts were recruited (T0). After enrollment, 5 patients with CE3b cysts received albendazole, resulting in cyst solidification (CE4) in 4/5. Within a two-year follow-up, the whole-blood test was repeated at two time-points, in ≥14 (T1) and in ≥4 (T2) patients per group. IL-4 and a panel of other soluble factors were measured in the stimulated plasma.

Baseline IL-4 levels were significantly higher in patients with CE3b compared to those with CE4 cysts (p = 0.006). Test accuracy for CE3b diagnosis had a sensitivity of 33–60% and a specificity of 76–95%, depending on the cut-off applied. Overall, IL-4 levels did not change significantly over time in either group; however, patients within the CE3b group showed a significant decrease of IL-1ra, IL-6, IL-8, G-CSF, IFN-γ, IP-10, MCP-1, MIP-1α, FGF at T1 compared to T0 (p≤0.042).

**Conclusions/Significance:**

Whole-blood IL-4-response to AgB is significantly higher in patients with active compared to inactive CE but apparently not modulated over time after treatment. On the contrary, the levels of IL-1ra, IL-6, IL-8, G-CSF, IFN-γ, IP-10, MCP-1, MIP-1α, FGF significantly decreased in active CE during follow-up. Additional studies are needed to understand whether these findings might have a clinical significance for patients’ follow-up.

## Author summary

Cystic echinococcosis (CE) is a complex disease for which a clear understanding of clinical manifestations is needed to avoid misdiagnosis, inappropriate treatment, and severe complications. The development of a laboratory test able to assess CE cyst viability and reactivation over time would facilitate the classification of patients and cysts, their rational assignment to a clinical management option and the implementation of a regular follow-up. Here, we evaluated the accuracy of a cytokine-based whole-blood test to detect the reactivation and the viability of hepatic CE cysts. The results showed that IL-4-response to AgB is not modulated over-time after treatment, while, IL-1ra, IL-6, IL-8, G-CSF, IFN-γ, IP-10, MCP-1, MIP-1α, FGF levels significantly decreased in active CE during follow-up. Additional studies are needed to understand whether these findings might have a clinical significance for patients’ follow-up.

## Introduction

Cystic echinococcosis (CE) is a parasitic zoonosis globally distributed, caused by infection with the larval stage (cysts) of *Echinococcus granulosus* sensu lato, endemic in rural livestock-raising areas, including Italy [1]. Human CE has considerable medical and economic impact, causing the loss of estimated >1 million disability adjusted life years (DALYs) every year [2]. CE is a complex disease with clinical presentations ranging from asymptomatic infection to severe, even fatal, disease [3]. CE cysts develop most commonly in the liver, followed by lungs, but all organs/tissues may be affected. Furthermore, CE cysts may present in different stages, each with a peculiar morphology and different recommended clinical management approach [4]. Inaccurate diagnosis and staging contribute to the health and economic burden that CE causes on patients and health systems in endemic areas. Currently, the gold standard for abdominal CE diagnosis and staging is ultrasound (US) [4], whereas serology, due to its limitations [5,6], can only support the diagnosis in cases where imaging is inconclusive [4].

US allows cyst classification into CE1, CE2, CE3a, CE3b, CE4, and CE5 stages [4]. This classification reflects the different “activity” of cyst stages, with cysts in CE1, CE2 and CE3b stages being active, CE3a transitional, and CE4-CE5 inactive [7]. Cysts that reach the CE4 stage as the consequence of therapy have a higher reactivation rate compared to those that became inactive spontaneously. Thus, inactive cysts can reactivate [8–11], calling for a long-term (5–10 years) US follow-up. However, US requires specific expertise, not always available in rural areas where the disease is endemic. The development of a laboratory test able to assess cyst viability would facilitate the classification of patients and cysts, and their rational assignment to a clinical management option, reducing risks and costs of overtreatment of inactive cysts [12]. It would also improve the implementation of a systematic patient’s follow-up in areas where access to imaging and specific expertise is unavailable, allowing a “triage” of patients requiring referral to specialized centers.

Cytokine-release tests based on the stimulation of whole-blood *in vitro* are currently used for the diagnosis of latent tuberculosis [13–15] and have been explored in several infectious diseases such as hepatitis B [16], toxoplasmosis [17], cytomegalovirus infection [18], and COVID-19 [19]. An interleukin (IL)-4-release assay based on the *in vitro* stimulation of whole-blood with an enriched fraction of Antigen B (AgB) of *E*. *granulosus* or its peptides, has been set-up and preliminarily evaluated for CE diagnosis, showing potential to distinguish patients with active and inactive CE cysts [20–22]. Furthermore, several works described associations between cytokine profiles and CE clinical features [23–28]. In this prospective study, we explored the accuracy of the cytokine-release whole-blood test in discriminating cyst viability and in detecting cyst reactivation over time in patients with active CE3b and spontaneously-inactivated cysts.

## Methods

### Ethics statement

The Ethics Committees of IRCCS San Matteo Hospital Foundation, Pavia, Italy (N. 20150018938) and of Istituto Nazionale per le Malattie Infettive (INMI) “Lazzaro Spallanzani”-IRCCS, Roma, Italy, (N. 28/2014) approved the study.

### Study population

Patients with hepatic CE cysts in care at the outpatient clinic of San Matteo Hospital, who signed the informed written consent, were prospectively enrolled from September 2015 to September 2017. CE cysts were diagnosed and staged based on the presence of pathognomonic features on US, according to the WHO-Informal Working Group on Echinococcosis classification [4]. Two study groups were defined: the “CE3b-group” and the “CE4-group”.

Inclusion criteria for the “CE3b-group” were presence of ≥1 hepatic CE cyst in CE3b stage, regardless of previous treatment, or presence of ≥1 hepatic CE cyst in CE4 stage resulting from the albendazole treatment of a CE3b cyst. Patients with cysts in this latter condition were included in the “CE3b-group” because, in the vast majority of cases, CE4 cysts resulting from albendazole treatment of CE3b cysts are only temporarily inactive, and reactivate to CE3b on average 6–12 months after inactivation [9]. The exclusion criterion for the CE3b-group was the presence of concomitant (hepatic or extrahepatic) cyst in CE1, CE2, or CE3a stage.

The inclusion criterion for the “CE4-group” was the presence of ≥1 hepatic CE cyst in CE4 or CE5 stage, spontaneously-inactivated. Exclusion criteria for CE4-group were the presence of concomitant (hepatic or extrahepatic) cysts in CE1, CE2, CE3a, or CE3b stages and having received previous treatment.

Demographic and clinical information were collected at enrollment (baseline-T0); blood samples for routine serology and for the experimental whole-blood test were collected at baseline and at two consecutive follow-up time points (T1 and T2) over two years. Routine serology tests performed at San Matteo Hospital Foundation were RIDASCREEN Echinococcus IgG ELISA (R-Biopharm, Darmstadt, Germany), Cellognost Echinococcosis indirect hemagglutination (IHA; Siemens, Erlangen, Germany), Western Blot Echinococcus WB IgG (LDBIO Diagnostics, Lyon, France), and VIRapid HYDATIDOSIS immunochromatographic test (ICT; Vircell, Granada, Spain).

### Whole-blood test

Five-hundred microliters of heparinized whole-blood in duplicate were stimulated with 1 μg/mL AgB-enriched fraction [20–22], 200 ng/mL staphylococcal enterotoxin B (SEB–stimulation control), or left unstimulated, and incubated overnight at 37°C, 5% CO_2_. Supernatants were then harvested, frozen and sent to INMI for cytokine analysis.

### Cytokine analysis

For all determinations, the laboratory staff was blinded to the sample classification. IL-4 determination was performed by high-sensitive ELISA (Quantikine HS IL-4 ELISA, R&D Systems, Minneapolis, MN, USA), according to manufacturer’s instructions. IL-4 range of detection was 0.25–16 pg/mL. The plasma leftover from IL-4 determination was tested for immune/growth factors [IL-1β, IL-1 receptor antagonist (ra), IL-2, IL-4, IL-5, IL-6, IL-7, IL-8, IL-9, IL-10, IL-12p70, IL-13, IL-15, IL-17A, eotaxin, fibroblast growth factor (FGF), granulocyte-colony stimulating factor (G-CSF), granulocyte-macrophage colony-stimulating factor (GM-CSF), interferon (IFN)-γ, IFN-γ-induced protein 10 (IP-10), monocyte chemoattractant protein-1 (MCP-1), macrophage inflammatory protein (MIP)-1α, MIP-1β, platelet-derived growth factor (PDGF), RANTES (regulated on activation, normal T cell expressed and secreted), tumour necrosis factor-α (TNF-α), vascular endothelial growth factor (VEGF)] using Bio-Plex Pro Human Cytokine 27-plex Assay panel and the MagPix system (all from Bio-Rad, Hercules, CA, USA), as per manufacturer’s instructions. Data were analyzed as previously described [19]. Briefly, values below or above the detection limits were converted to 0 or to the highest value of the standard curve, respectively. Values originated from ≥50 beads-reading were included. In all cases, values of each sample were subtracted from those of the unstimulated control.

### Statistical analysis

The analysis was carried out with Prism 8 software (Graphpad Software 8.0, San Diego, MO, USA). Medians and interquartile ranges (IQR) were calculated for continuous measures. Groups were compared using Mann–Whitney U, Wilcoxon, and Chi-square tests, as appropriate. Receiver Operator Characteristic (ROC) analysis was used to define cut-off values and sensitivity/specificity of the test; Spearman Rank Correlation was applied for correlations (r_s_>0.7 was considered high correlation, 0.7<r_s_>0.5 moderate correlation and r_s_<0.5 low correlation).

## Results

### Study population

We enrolled 30 patients within the CE3b-group and 37 within the CE4-group. Their demographic and clinical information are shown in Table 1.

**Table 1:** Demographic and clinical characteristics of the enrolled subjects.

	CE3b-group	CE4-group	P value
**N (%)**	30 (100.0)	37 (100.0)	
**Median age—years (IQR)**	52 (40–63)	54 (44–65)	0.55*
**Female sex—N (%)**	13 (43.3)	28 (75.7)	**0.007** ^**#**^
**Origin—N (%)**			0.1^**#**^
**Italy**	15 (50.0)	26 (70.3)	
**Eastern Europe**	8 (26.7)	6 (16.2)	
**Africa**	7 (23.3)	3 (8.1)	
**South America**	-	2 (5.4)	
**Serology positive results—N (%)**	24 (80.0)	6 (16.2)	-
**Previous treatment—N (%)**	21 (70.0)	0 (0)	-
**Years from last treatment—median (IQR)**	3 (1–4)	-	-
**Current treatment—N (%)**	6 (20.0)	0 (0)	-
**Number of study-target cysts—median (IQR)**	1 (1–1)	1 (1–1)	0.29*
**Number of total cysts—median (IQR)**	1 (1–2)	1 (1–2)	0.08*

**Footnote**: N: Number; IQR: Interquartile Range.

* Mann–Whitney U test

^#^ Chi-square test.

Within the CE3b-group (n = 30), at enrollment 26 patients had CE3b cysts and 4 had treatment-inactivated CE4 cysts deriving from CE3b cysts, with the last treatment dating back 18 months before enrollment (median: 18 months; IQR: 12–33 months). Fifteen patients were evaluated at T1 (median time after enrollment: 6 months; IQR: 6–12 months). After enrollment, albendazole was administered to 5 patients with CE3b cysts, which induced cysts solidification (CE4 stage) in 4 of them, and therapy started before enrollment was continued in one patient who showed treatment-induced CE4 at baseline. This patient showed reactivation (to CE3b) of the cyst at T1. The patients not receiving albendazole at enrollment did not show any change of the cyst stage at T1. The second follow-up (T2) was performed in 5 patients (median time after enrollment: 24 months; IQR: 15–24 months). None of them received albendazole between T1 and T2. The patient showing the transition from albendazole-induced CE4 at T0 to CE3b at T1 received surgical resection of the cyst between T1 and T2, and was therefore excluded from the T2 analysis. At T2, 3/5 patients showed a reactivation of the cysts compared to T1.

Within the CE4-group, 23 patients had spontaneously-inactivated CE4 cysts, 11 had spontaneously-inactivated CE5 cysts and 3 had cysts in both stages. Fourteen patients were evaluated at T1 (median time after enrollment: 12 months; IQR: 12–20 months). The second follow-up (T2) was performed in 4 patients (median time after enrollment: 24 months; IQR: 15–24). No change in cyst stage was observed in this group over time, and no patient received treatment.

### The AgB-specific IL-4-response is significantly higher in the presence of CE3b cysts

**Fig 1:**
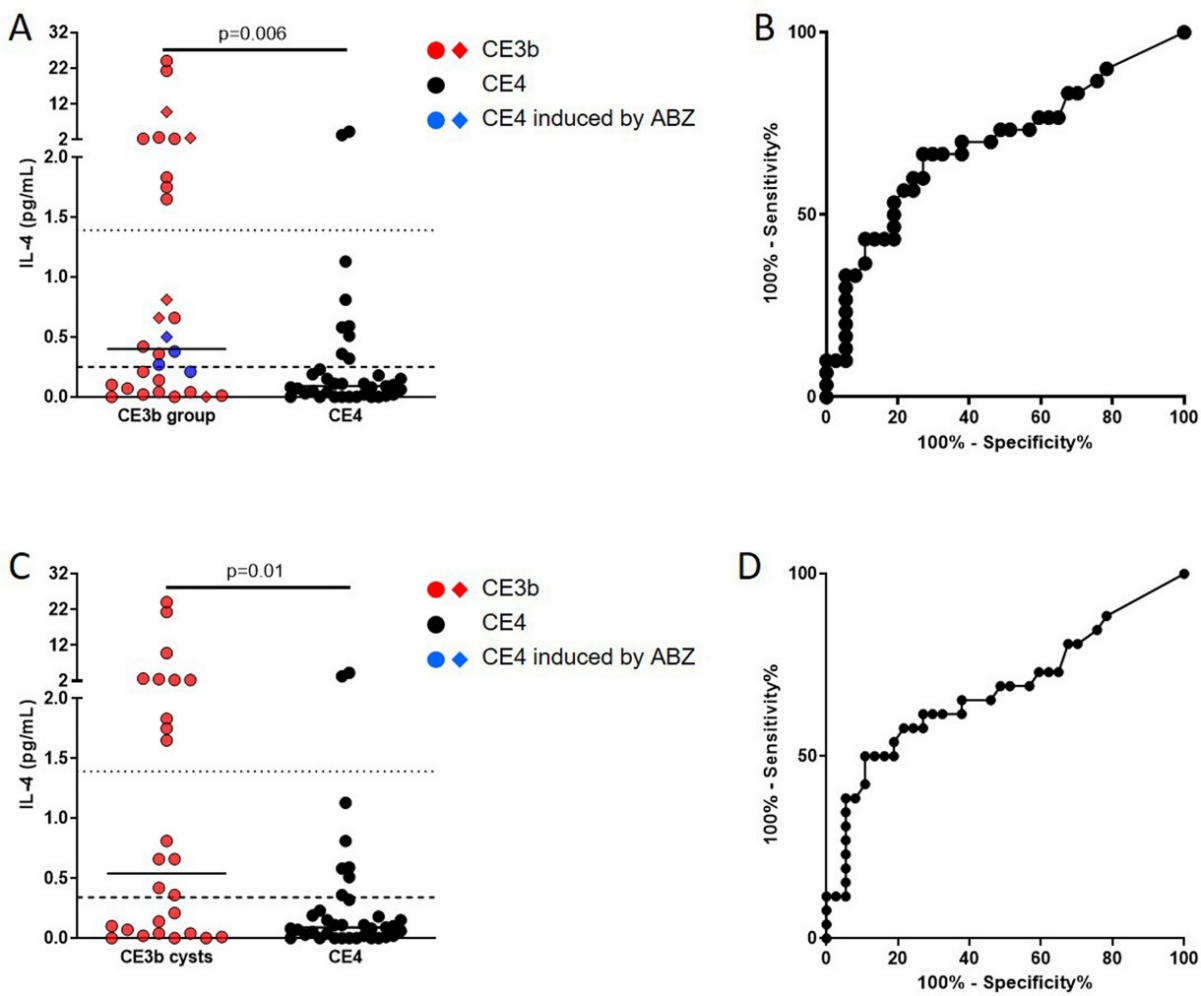
IL4-specific response is significantly associated to CE3b cysts. **A.** IL-4 levels are significantly increased in the CE3b-group compared to CE4-group. **B.** AUC analysis for CE3b-group diagnosis; the 0.25 pg/mL cut-off maximizing sensitivity for CE3b-group diagnosis (panel A, dashed line) showed a 60% sensitivity and 76% specificity whereas the 1.39 pg/mL cut-off to maximize specificity (panel A, dotted line) showed 33% sensitivity and 95% specificity. **C.** The IL-4 levels were significantly increased in patients with CE3b cysts compared to the CE4-group. **D.** AUC analysis for CE3b diagnosis; the cut-off of 0.34 pg/mL maximizing sensitivity for CE3b cysts (panel C, dashed line) predicted CE3b with a 57.7% sensitivity and 78.4% specificity; the cut-off of 1.39 pg/mL cut-off to maximize specificity (panel C, dotted line) predicted CE3b with 38.5% sensitivity and 95% specificity. **Footnotes:** Horizontal bars represent medians. IL-4 concentrations were determined by ELISA. Responses in panels A and C were compared using the Mann-Whitney test; ROC-analysis was applied in panels B and D; differences were considered significant at p-values of ≤0.05. Red dots indicate patients with CE3b cysts, blue dots indicate patients with treatment-induced CE4 cysts, black dots indicate patients with spontaneously-inactivated cysts, diamond symbols indicate that the patient received albendazole. IL: Interleukin; CE: Cystic Echinococcosis; ABZ: Albendazole.

IL-4 levels at baseline were significantly higher in the CE3b-group compared to the CE4-group (p = 0.006) (Fig 1A).

Moreover, a low but significant positive correlation (r_s_ = 0.43, p = 0.02) was found in patients in the CE3b-group between the IL-4 levels and the total number of CE cysts of each patient (S1 Fig). IL-4 levels were compared at baseline among patients with CE3b cysts (n = 26 in the CE3b-group), with treatment-induced CE4 cysts (n = 4 in the CE3b-group) and with spontaneously-inactivated cysts (n = 37; CE4-group). The highest IL-4 levels were found in patients with CE3b cysts compared to the other groups (IL-4 medians: CE3b 0.54 pg/mL> treatment-induced CE4 0.33 pg/mL> spontaneously-inactivated cysts 0.09 pg/mL) and the difference was significant comparing patients with CE3b cysts and patients with spontaneously-inactivated cysts (p = 0.014) (S2 Fig).

To explore the correlation between IL-4-response and the humoral response, patients were stratified based on serology (excluding patients with discordant serology results, n = 4). In the CE3b-group, a higher IL-4-response to AgB was observed in sero-positive compared to sero-negative patients (S3A Fig); no differences were found between patients in the CE4-group with positive or negative serology (S3B Fig).

A ROC analysis was performed for CE3b-group diagnosis (Fig 1B). Significant area under curve (AUC) results were obtained (AUC, 0.69; 95% confidence interval (CI), 0.56–0.82, p = 0.007). The 0.25 pg/mL cut-off maximizing sensitivity for CE3b-group diagnosis (Fig 1A, dashed line) showed 60% sensitivity (95% CI, 40.6%-77.3%) and 76% specificity (95% CI, 58.8%-88.2%) whereas the 1.39 pg/mL cut-off to maximize specificity (Fig 1A, dotted line) showed 33% sensitivity (95% CI, 17.3%-52.8%) and 95% specificity (95% CI, 81.8%-99.3%). When excluding from the analysis patients within the CE3b-group showing treatment-induced CE4 cysts at T0 (n = 4), results were comparable (Fig 1C and 1D). Indeed, ROC analysis for diagnosing CE3b cysts showed a significant AUC result (AUC = 0.68; 95% CI, 0.54–0.82, p = 0.014) (Fig 1D). The cut-off of 0.34 pg/mL maximizing sensitivity for CE3b cysts (Fig 1C, dashed line) predicted CE3b with 57.7% sensitivity (95% CI, 36.9%-76.7%) and 78.4% specificity (95% CI, 61.8%-90.2%); the cut-off of 1.39 pg/mL cut-off to maximize specificity (Fig 1C, dotted line) predicted CE3b with 38.5% sensitivity (95% CI, 20.2%-59.4%) and 95% specificity (95% CI, 81.8%-99.3%).

### In the CE3b-group, the AgB-specific IL-4-response changed over time compared to baseline independently of therapy

After enrollment, the whole-blood test was repeated at T1 and T2 in 15 and 5 patients in the CE3b-group and in 14 and 4 patients in the CE4-group, respectively.

**Fig 2:**
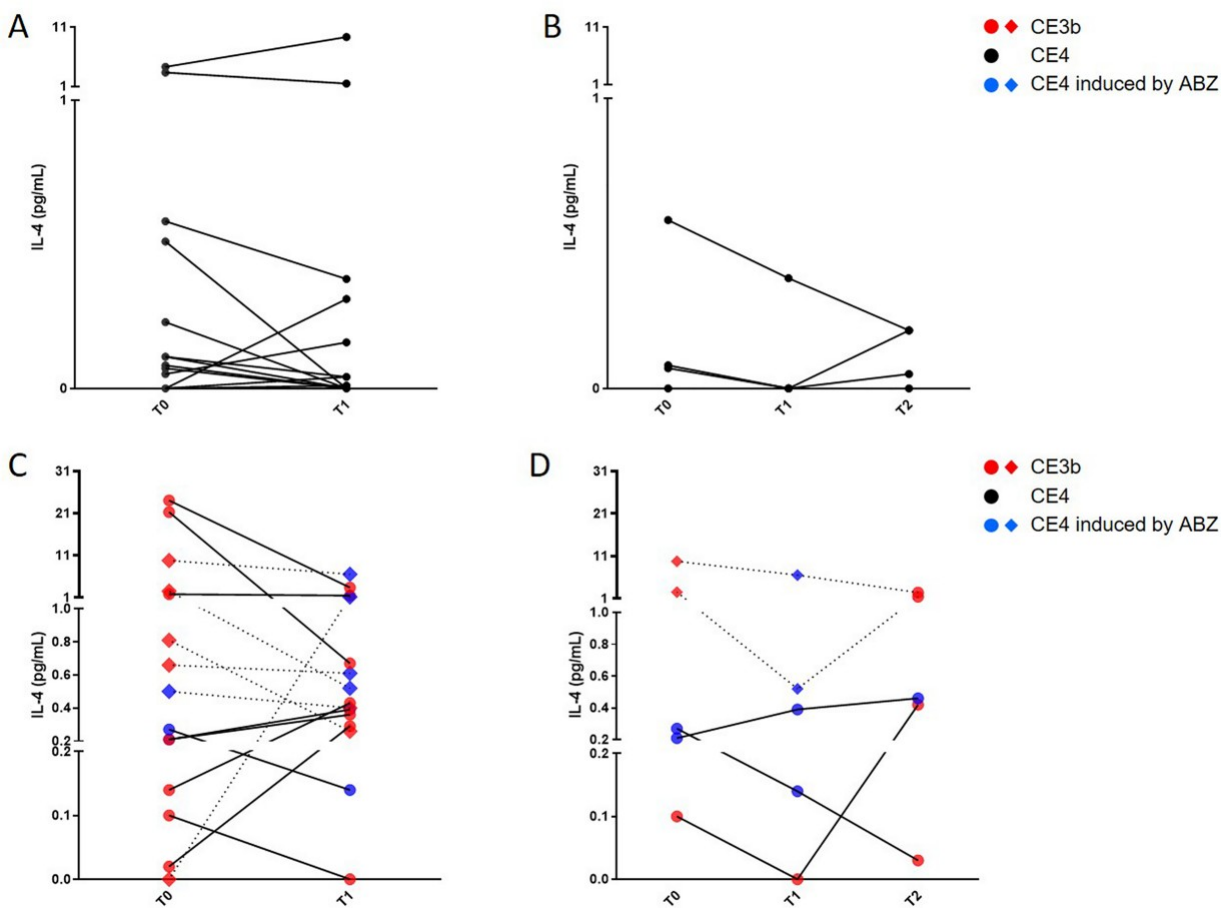
In the CE3b-group, the AgB-specific IL-4 response changed over time independently of therapy. Whole-blood test analysis at T1 (15 patients in the CE3b-group and in 14 patients in the CE4-group) and at T2 (5 patients in the CE3b-group and in 4 patients in the CE4-group). **A.** Analysis of IL-4 levels in the CE4-group at T1 compared to T0. **B.** Analysis of IL-4 levels in the CE4-group at T2 compared to T0 or T1. **C.** Analysis of IL-4 levels in the CE3b-group at T1 compared to T0. IL-4 levels decreased in 6 patients, increased in 2, and were unchanged in 7 patients at T1 compared to T0. The treatment led to cyst solidification (CE4 at T1) in 4/5 of patients with CE3b at T0: 2 patients showed a IL-4 decline; one patient showed almost no change and one patient showed an increase. **D.** Analysis of IL-4 levels in the CE3b-group at T2 compared to T0 or T1. At T2, 3/4 with CE4 cysts at T1 had their cysts reactivated to CE3b stage and IL-4 levels increased at T2 only in 1 of them. **Footnotes:** IL-4 concentrations were determined by ELISA. Responses in all the panels were compared using the Wilcoxon test; differences were considered significant at p-values of ≤0.05. Red dots indicate patients with CE3b cysts, blue dots indicate patients with treatment-induced CE4 cysts, black dots indicate patients with spontaneously-inactivated cysts, diamond symbols indicate that the patient received albendazole.The continuous lines indicate that no treatment was administered; dotted lines indicate albendazole between the 2 time points. IL: Interleukin; CE: Cystic Echinococcosis; ABZ: Albendazole.

As expected, we observed no change in IL-4 levels in the CE4-group at T1 compared to T0 (p = 0.38) with the exception of 4 patients, 3 of whom showed a ≥34% decrease and 1 an increase of 118% (Fig 2A).

IL-4 levels at T2 did not significantly change (p = 0.43) with the exception of 1 patient who showed a 66% decrease from T0 to T2 (Fig 2B).

Regarding the CE3b-group, the IL-4 levels at T1 compared to T0 decreased in 6 patients, increased in 2, and were unchanged in 7 patients. Overall, no significant differences were found between the two time points (p = 0.2). Five patients with CE3b cysts (red diamonds in Fig 2C and 2D) and one patient with treatment-induced CE4 stage (blue diamonds in Fig 2C and 2D) were receiving albendazole at enrollment (dotted lines in Fig 2C). The treatment led to cyst solidification (CE4 at T1) in 4/5 of patients with CE3b at T0. Within these 4 subjects with therapy-induced CE4 cysts at T1, compared to T0 (Fig 2C), 2 patients showed a ≥33% IL-4 decline; one patient showed almost no change (7.6% decrease) and one patient showed an increase from 0 to 1.16 pg/mL. At T2, 3 of the 4 patients with CE4 cysts at T1 had their cysts reactivated to CE3b stage (red dots in Fig 2D) and the analysis of the IL-4 levels showed an increase only in 1 of them (Fig 2D). We also evaluated the IL-4-response stratifying patients in the CE3b-group at T1 as follows: patients not receiving albendazole at T0 (n = 7), still showing CE3b cysts at T1 after receiving albendazole at T0 (n = 2), and showing treatment-induced CE4 cysts at T1 after receiving albendazole at T0 (n = 4). The two patients with treatment-induced CE4 cysts at T0 in the CE3b-group were excluded. Although no significant differences were found among the three subgroups, the highest IL-4 levels were found in patients with treatment-induced CE4 cysts (S4 Fig).

### AgB-specific IL-1ra, IL-6, IL-8, FGF, G-CFS, IFN-γ, MIP-1α, IP-10 and MCP-1 levels in patients in the CE3b-group are significantly reduced over time compared to baseline

To assess if other factors may be useful to monitor cysts reactivation/inactivation, we evaluated a panel of immune/growth factors in the AgB-stimulated plasma. The analysis was performed in 12 patients per study group, from whom plasma was available after the IL-4 analysis. In the CE3b-group, 10 patients had CE3b cysts at T0 and 4/10 received albendazole at enrollment that induced solidification of the cysts in 3/4 patients at T1; 2 patients had treatment induced-CE4 cysts at T0 and in 1/2 the cyst reactivated at T1.

Although patients in the CE3b-group showed higher levels compared to patients in the CE4-group for the majority of the analytes considered, no significant differences were found between the two groups at baseline (Table 2 and S5 Fig).

**Table 2:** Levels of immune/growth factors in the CE3b- and CE4-groups.

	CE3b group			CE4 group			P value**
	T0	T1	p value*	T0	T1	p value*	
**IL-1β** median (IQR)	2.7 (0.1–37.3)	1.0 (0–6.4)	0.2754	0.1 (0–8.8)	0 (0–0.6)	0.3828	0.3031
**IL-1ra** median (IQR)	671.4 (163.4–5068.0)	124.3 (0–1283.0)	**0.0269**	330.2 (37.0–2495.0)	44.4 (0–669.5)	**0.0273**	0.3685
**IL-2** median (IQR)	2.9 (11.7–521.7)	7.8 (0.2–24.4)	0.0830	3.9 (0.3–94.1)	1.6 (0–44.1)	>0.9999	0.2392
**IL-4** median (IQR)	0.6 (0–17.9)	0 (0–2.6)	0.1484	0.5 (0–8.3)	0.1 (0–2.5)	0.4922	0.7178
**IL-5** median (IQR)	13.9 (1.9–96.0)	3.6 (0–22.4)	0.1641	1.9 (0–32.9)	1.2 (0–6.9)	0.6250	0.3084
**IL-6** median (IQR)	8.8 (1.4–236.2)	0.9 (0–47.1)	**0.0420**	3.4 (0–35.6)	1.9 (0–21.1)	0.6953	0.2095
**IL-7** median (IQR)	0 (0–0)	0 (0–2.7)	>0.9999	0 (0–0)	0 (0–3.3)	0.6250	0.8634
**IL-8** median (IQR)	650.7 (101.3–2017.0)	68.7 (0–639.3)	**0.0098**	78.3 (0–262.7)	34.3 (0–362.3)	0.1475	0.0671
**IL-9** median (IQR)	14.5 (8.4–192.3)	13.2 (0–34.7)	0.1475	1.7 (19.5–59.5)	0 (0–18.0)	0.4316	0.7431
**IL-10** median (IQR)	1.2 (0.1–3.1)	0.3 (0–3.9)	0.5771	1.5 (0.5–1.6)	0 (0–2.1)	0.5625	0.9885
**IL-12** median (IQR)	0.3 (0–7.6)	0 (0–5.4)	0.7422	0.3 (0–2.5)	0 (0–0.5)	0.8438	0.8734
**IL-13** median (IQR)	2.7 (0–117.6)	0.1 (0–9.2)	0.1953	0 (0–18.4)	0 (0–16.9)	0.8125	0.3253
**IL-15** median (IQR)	0 (0–559.1)	0 (0–169.1)	0.1563	0 (0–185.0)	0 (0–125.3)	>0.9999	0.6913
**IL-17** median (IQR)	3.8 (1.2–72.4)	1.9 (0–9.6)	0.1182	1.8 (0–27.8)	0 (0–9.7)	0.9102	0.4166
**EOTAXIN** median (IQR)	3.7 (2.9–12.3)	0 (0–8.7)	0.1055	2.6 (0–5.6)	2.1 (0–5.4)	0.6250	0.0962
**FGF** median (IQR)	23.0 (10.5–152.1)	2.2 (0–32.9)	**0.0195**	13.8 (0–46.8)	0 (0–12.3)	0.4922	0.2434
**G-CSF** median (IQR)	231.5 (108.0–3892.0)	46.1 (0–565.8)	**0.0161**	63.2 (3.5–685.0)	31.8 (0–459.0)	>0.9999	0.1132
**GM-CSF** median (IQR)	1.6 (0.5–20.6)	0.6 (0–4.6)	0.1309	1.1 (0.1–6.9)	0.6 (0–4.1)	0.8457	0.3674
**IFN-γ** median (IQR)	70.9 (29.9–397.3)	17.9 (0–108.3)	**0.0093**	17.9 (6.3–186.5)	9.2 (0.3–81.7)	0.3013	0.1245
**IP-10** median (IQR)	188.6 (1.7–567.5)	0 (0–75.6)	**0.0059**	75.3 (0–443.9)	22.3 (0–256.8)	0.3223	0.7520
**MCP-1** median (IQR)	817.2 (165.0–3254.0)	99.4 (7.85–834.5)	**0.0024**	313.1 (54.2–1904.0)	64.9 (7.5–629.4)	0.0640	0.3859
**MIP-1α** median (IQR)	3.8 (1.6–150.9)	2.2 (0–14.3)	**0.0420**	1.5 (0.1–20.3)	1.7 (0–9.5)	0.7646	0.2016
**PDGF** median (IQR)	12.9 (0–104.4)	0 (0–50.2)	0.2500	20.2 (0–56.9)	2.2 (0–27.9)	0.1602	0.9767
**MIP-1β** median (IQR)	45.6 (0–1650.0)	26.1 (0–275.6)	0.0645	94.9 (12.6–618.2)	23.8 (0–359.9)	0.3652	0.5983
**RANTES** median (IQR)	313.4 (0–817.6)	0 (0–621.0)	0.2031	30.8 (0–173.3)	0 (0–126.2)	0.7422	0.0801
**TNF-α** median (IQR)	20.2 (0.4–490.2)	5.9 (0–42.1)	0.0547	8.3 (0.4–107.1)	7.0 (0–47.6)	>0.9999	0.5968
**VEGF** median (IQR)	67.7 (0–290.8)	52.1 (0–107.7)	0.3013	54.4 (0.3–189.3)	36.5 (0.6–116.5)	0.8311	0.9771

Footnotes: IL: Interleukin; ra: receptor antagonist; FGF: fibroblast growth factor; G-CSF: granulocyte-colony stimulating factor; GM-CSF: granulocyte-macrophage colony-stimulating factor; IFN: Interferon; IP: Interferon gamma-induced protein; MCP: monocyte chemoattractant protein; MIP: macrophage inflammatory protein; PDGF: Platelet-derived growth factor; RANTES: regulated on activation. normal T cell expressed and secreted; TNF: tumour necrosis factor; VEGF: vascular endothelial growth factor.

* Wilcoxon test applied between T0 and T1 within each group.

** Mann-Whitney U test applied between CE3b T0 and CE4 T0.

**Fig 3:**
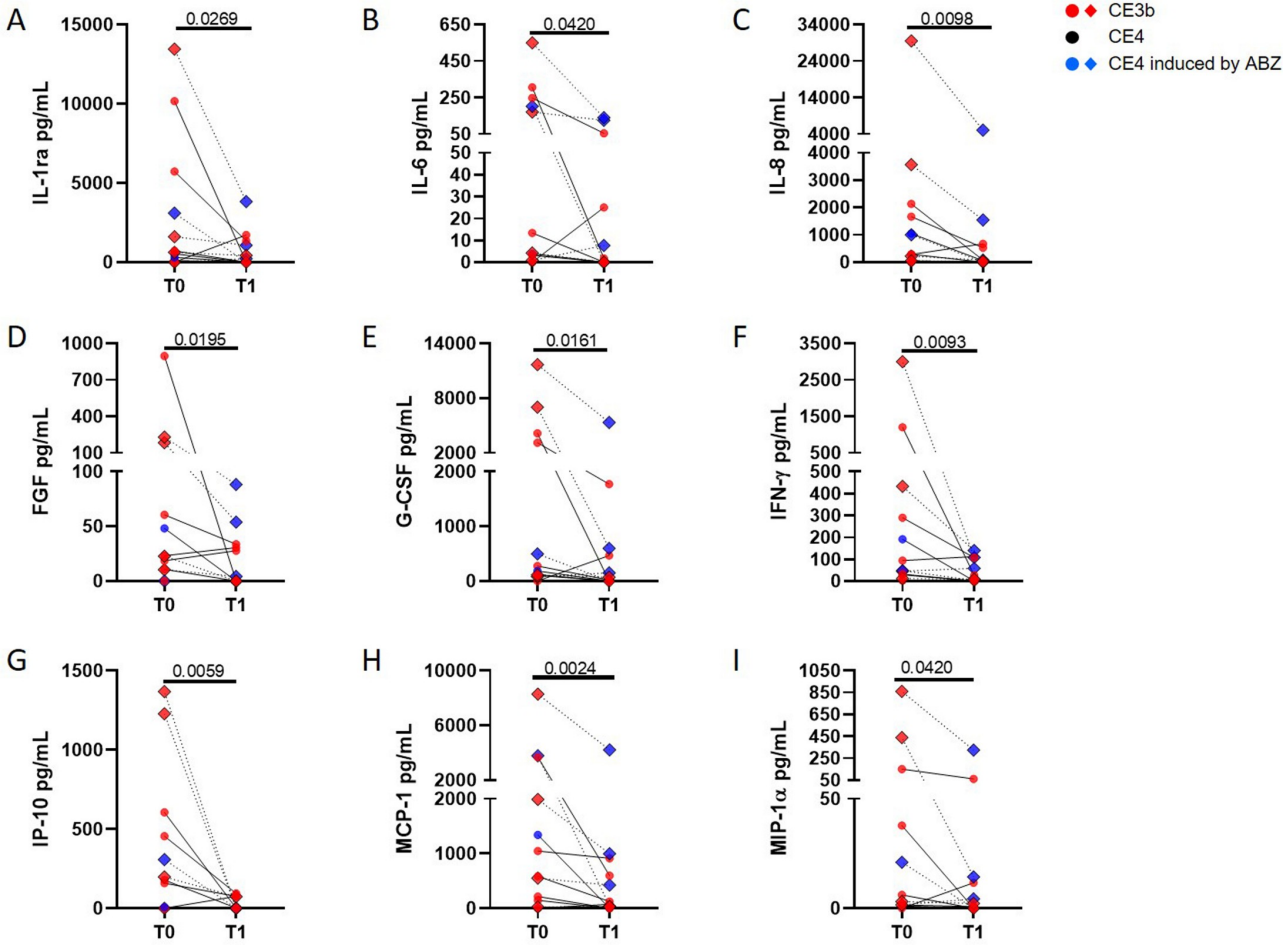
The AgB-specific IL-1ra, IL-6, IL-8, FGF, G-CFS, IFN-γ, MIP-1α, IP-10 and MCP-1 levels in patients in the CE3b-group are significantly reduced overtime. Multiplex analysis performed in 12 patients per study group. The following soluble factors were evaluated: IL-1β, IL-1ra, IL-2, IL-4, IL-5, IL-6, IL-7, IL-8, IL-9, IL-10, IL-12p70, IL-13, IL-15, IL-17A, Eotaxin, basic FGF, G-CSF, GM-CSF, IFN-γ, IP-10, MCP-1, MIP-1α, MIP-1β, PDGF, RANTES, TNF-α, VEGF. In patients in the CE3b-group a significant decrease of IL-1ra (**A**), IL-6 (**B**), IL-8 (**C**), FGF (**D**), G-CSF (**E**), IFN-γ (**F**), IP-10 (**G**), MCP-1 (**H**) and MIP-1α (**I**) at T1 compared to T0 was observed. **Footnotes:** Analytes concentrations were determined by luminex. Responses in all the panels were compared using the Wilcoxon test; differences were considered significant at p-values of ≤0.05. Red dots indicate patients with CE3b cysts, blue dots indicate patients with treatment-induced CE4 cysts, black dots indicate patients with spontaneously-inactivated cysts, diamond symbols indicate that the patient received albendazole. The continuous lines indicate that no treatment was administered; dotted lines indicate albendazole between the 2 time points. IL: Interleukin; ra: receptor antagonist; G-CSF: granulocyte-colony stimulating factor; IFN: Interferon; IP-10: IFN-γ-induced protein-10; MCP: monocyte chemoattractant protein; FGF: fibroblast growth factor; MIP: macrophage inflammatory protein.

When the modulation over time of factors was evaluated in patients in the CE3b-group, we observed a significant decrease of IL-1ra (Fig 3A, p = 0.0269), IL-6 (Fig 3B, p = 0.042), IL-8 (Fig 3C, p = 0.0098), FGF (Fig 3D, p = 0.0195), G-CSF (Fig 3E, p = 0.0161), IFN-γ (Fig 3F, p = 0.0093), IP-10 (Fig 3G, p = 0.0059), MCP-1 (Fig 3H, p = 0.0024), and MIP-1α (Fig 3I, p = 0.0420) at T1 compared to T0.

No other factors were modulated at T1 compared to baseline (S6 Fig and Table 2).

The same analysis was conducted in patients in the CE4-group. No significant differences were found between T1 and T0, with the exception of IL-1ra that decreased in 9/12 patients evaluated (S7 Fig and Table 2, p = 0.0273). A heatmap of all the factors evaluated in the CE3b and in the CE4 groups at T0 and at T1 is shown in the S8 Fig.

## Discussion

CE is a complex disease, although mostly benign, for which a clear understanding of clinical manifestations is needed to avoid misdiagnosis, inappropriate treatment, and severe complications [29]. The development of a laboratory test able to assess cyst viability and reactivation over time would facilitate the implementation of a regular follow-up of patients in areas where access to imaging and specific expertise is unavailable, allowing a “triage” of patients requiring referral to specialized centres. Serology has proven so far of limited use for this purpose [6,30–32]. In this study, we evaluated the accuracy of an experimental whole-blood stimulation test based on the detection of cytokines release, to reflect cyst viability and detect reactivation during a two-year follow-up. The results showed that IL-4 measurement is not informative enough of cyst viability and reactivation to be used in clinical practice. However, other soluble factors such as IL-1ra, IL-6, IL-8, FGF, G-CFS, IFN-γ, MIP-1α, IP-10 and MCP-1 are modulated during follow-up, suggesting their potential for monitoring cysts viability. No other cytokines of the large panel tested was found significantly different between groups and time points.

CE is characterized by the coexistence of Th1 and Th2 responses; the Th2 response has been correlated with disease susceptibility/chronicity and the Th1 response with protective immunity [33]. *E*. *granulosus*-specific T-cell clones from patients with active cysts showed a mixed Th1/Th2 profile whereas clones from patients with inactive cysts a Th1 profile [34]. Recently, the proteomic analysis of circulating exosomes from CE patients confirmed the involvement of the Th1-Th2 and regulatory T-cell compartments in active CE [35]. Moreover, we showed that AgB-specific cells producing IL-2/TNF-α/Th2-cytokines were associated with presence of active cysts [36]. Studies evaluating the *ex-vivo* levels of circulating cytokines in patients with hepatic CE gave conflicting results [37,38]. However, when explored after *in-vitro* stimulation, we showed that the IL-4-specific-response using a whole-blood platform associated with cyst activity [20,21]. In agreement with these studies, here we confirmed that higher levels of IL-4 were associated with the presence of active CE3b cysts compared to inactive CE4 cysts. Test accuracy for CE3b diagnosis had a sensitivity of 60% with a specificity of 76%, or a sensitivity of 33% with a specificity of 95%, depending on the cut-off applied. The application of a lab-based test even with such moderate accuracy may still have a role in rural endemic areas where access to US diagnosis may be difficult, to refer for US evaluation those patients who might have active cysts, because CE3b cysts tend to remain stable over time [9] and do not generally need emergency treatment but rather careful planning of the best clinical management. Unfortunately, a stable trend of the cytokine response over time according to cyst evolution could not be detected, possibly due to a too short follow-up, and too few patients presented at T1 and T2 to properly estimate the usefulness of the test for follow-up.

When we explored other soluble factors, we found that their levels were not different at T0 between patients in the CE3b- and in the CE4-groups. However, in patients in the CE3b-group, IL-1ra, IL-6, IL-8, FGF, G-CFS, IFN-γ, MIP-1α, IP-10 and MCP-1 significantly decreased during follow-up.

Interestingly, some of these factors, as IL-8, MCP-1, IL-6, MIP-1α, G-CFS, are linked to neutrophils recruitment and differentiation. Neutrophils are key actors during infections as they mediate the resolution of the inflammation. Their role in CE is intriguing. AgB inhibits neutrophils chemotaxis [39]; moreover, protoscoleces, but not AgB, have been reported to induce neutrophils activation and increase IL-8 production [40]. If protoscoleces are accidentally released from cysts, their mediated activation of neutrophils is modulated by AgB, possibly allowing the development of secondary cysts [39]. Therefore, the reduction of neutrophil-associated factors during follow-up in patients with CE3b cysts may have the dual interpretation of deriving from inflammation resolution or, more likely, being the result of a negative immunomodulatory effect mediated by AgB. These findings need to be confirmed in larger studies to establish associations with inactivation/reactivation.

This study had several limitations, including the low number of patients fulfilling the inclusion criteria, the short period of the study, and the small number of patients who attended the follow-up visits. A future study should envisage a longer recruitment period and follow-up length to guarantee the inclusion of an adequate sample size to confirm our findings. On the other hand, this study had the strength of the prospective design and of the stringent characterization of the enrolled population.

To conclude, we showed that the *in-vitro* IL-4-specific-response measurement in whole-blood, although associated with cyst viability, is probably not informative enough of the viability and reactivation of the cyst to be used in clinical practice, although a clear valuation of assay performance for patients’ follow-up could not be performed. Interestingly, the levels of other soluble factors such as IL-1ra, IL-6, IL-8, FGF, G-CFS, IFN-γ, MIP-1α, IP-10 and MCP-1 did change during follow-up, encouraging the investigation of a panel of immune/growth factors in a larger population and for a longer follow-up time length.

## Supporting information

S1 Fig Low but significant positive correlation in the CE3b-group between the IL-4 levels and the number of total echinococcal cysts. A low but significant positive correlation was found in patients in the CE3b-group between the IL-4 levels and the number of total echinococcal cysts of each patient. **Footnotes:** IL-4 concentrations were determined by ELISA. Analysis was conducted using Spearman Rank Correlation for correlations (r_s_>0.7 was considered high correlation, 0.7<r_s_>0.5 moderate correlation and r_s_<0.5 low correlation); differences were considered significant at p-values of ≤0.05. IL: Interleukin.
(TIF)

S2 Fig At baseline, patients with CE3b cysts show the highest IL-4 levels compared to patients with treatment-induced CE4 cysts and with spontaneously-inactivated cysts. IL-4 levels compared at baseline among patients with CE3b cysts (n = 26 in the CE3b-group), with treatment-induced CE4 cysts (n = 4 in the CE3b-group) and with spontaneously-inactivated cysts (n = 37; CE4-group). The highest IL-4 levels were found in patients with CE3b cysts compared to the other groups. **Footnotes:** Horizontal bars represent medians. IL-4 concentrations were determined by ELISA. Responses were compared using the Mann-Whitney test with Bonferroni correction; differences were considered significant at p-values of ≤0.016. IL: Interleukin; CE: Cystic Echinococcosis.
(TIF)

S3 Fig A higher IL-4 response to AgB associate to CE3b-group with a positive serology compared to those with negative serology. Analysis of IL-4 levels in response to AgB at baseline stratifying the patients based on serology. **A.** A higher IL-4 response to AgB was observed in patients in the CE3b-group with a positive serology compared to those with negative serology. **B.** no differences in patients in the CE4-group. **Footnotes:** Horizontal bars represent medians. IL-4 concentrations were determined by ELISA. Responses in all the panels were compared using the Mann-Whitney; differences were considered significant at p-values of ≤0.05. IL: Interleukin; CE: Cystic Echinococcosis.
(TIF)

S4 Fig Patients with treatment-induced CE4 cysts show the highest IL-4 levels at T1. Comparison of IL-4-response comparing patients in the CE3b-group at T1 as follows: patients not receiving albendazole at T0 (n = 7), still showing CE3b cysts at T1 after receiving albendazole at T0 (n = 2), and showing treatment-induced CE4 cysts at T1 after receiving albendazole at T0 (n = 4). The highest IL-4 levels were found in patients with treatment-induced CE4 cysts. **Footnotes:** Horizontal bars represent medians. IL-4 concentrations were determined by ELISA. Responses were compared using the Mann-Whitney test with Bonferroni correction; differences were considered significant at p-values of ≤0.016. IL: Interleukin; CE: Cystic Echinococcosis.
(TIF)

S5 Fig No differences between CE3b-group and CE4-group at baseline for all the soluble factors evaluated by luminex. The following soluble factors were evaluated: IL-1β, IL-1ra, IL-2, IL-4, IL-5, IL-6, IL-7, IL-8, IL-9, IL-10, IL-12p70, IL-13, IL-15, IL-17A, Eotaxin, basic FGF, G-CSF, GM-CSF, IFN-γ, IP-10, MCP-1, MIP-1α, MIP-1β, PDGF, RANTES, TNF-α, VEGF. Although patients in the CE3b-group showed higher levels compared to patients in the CE4-group for the majority of the analytes considered, no significant differences were found between the two groups at baseline. **Footnotes:** Horizontal bars represent medians. Analytes concentrations were determined by luminex. Responses in all the panels were compared using the Mann-Whitney; differences were considered significant at p-values of ≤0.05. IL: Interleukin; ra: receptor antagonist; FGF: fibroblast growth factor; G-CSF: granulocyte-colony stimulating factor; GM-CSF: granulocyte-macrophage colony-stimulating factor; IFN: interferon; IP: IFN-γ-induced protein; MCP: monocyte chemoattractant protein; MIP: macrophage inflammatory protein; PDGF: Platelet-derived growth factor; RANTES: regulated on activation, normal T cell expressed and secreted; TNF: tumour necrosis factor; VEGF: vascular endothelial growth factor.
(TIF)

S6 Fig Analysis of soluble factors in CE3b-group during follow-up. Multiplex analysis performed in 12 patients per study group. The following soluble factors were evaluated: IL-1β, IL-2, IL-4, IL-5, IL-7, IL-9, IL-10, IL-12p70, IL-13, IL-15, IL-17A, Eotaxin, GM-CSF, MIP-1β, PDGF, RANTES, TNF-α, VEGF. None of these factors showed were modulated at T1 compared to baseline. **Footnotes:** Analytes concentrations were determined by luminex. Responses in all the panels were compared using the Wilcoxon test; differences were considered significant at p-values of ≤0.05. IL: Interleukin; GM-CSF: granulocyte-macrophage colony-stimulating factor; MIP: macrophage inflammatory protein; PDGF: Platelet-derived growth factor; RANTES: regulated on activation, normal T cell expressed and secreted; TNF: tumour necrosis factor; VEGF: vascular endothelial growth factor.
(TIF)

S7 Fig Analysis of soluble factors in CE4-group during follow-up. Multiplex analysis performed in 12 patients per study group. The following soluble factors were evaluated: IL-1β, IL-1ra, IL-2, IL-4, IL-5, IL-6, IL-7, IL-8, IL-9, IL-10, IL-12p70, IL-13, IL-15, IL-17A, Eotaxin, basic FGF, G-CSF, GM-CSF, IFN-γ, IP-10, MCP-1, MIP-1α, MIP-1β, PDGF, RANTES, TNF-α, VEGF. No factors, excepted IL-1ra, were modulated at T1 compared to baseline. **Footnotes:** Analytes concentrations were determined by luminex. Responses in all the panels were compared using the Wilcoxon test; differences were considered significant at p-values of ≤0.05. IL: Interleukin; ra: receptor antagonist; FGF: fibroblast growth factor; G-CSF: granulocyte-colony stimulating factor; GM-CSF: granulocyte-macrophage colony-stimulating factor; IFN: interferon; IP: IFN-induced protein; MCP: monocyte chemoattractant protein; MIP: macrophage inflammatory protein; PDGF: Platelet-derived growth factor; RANTES: regulated on activation, normal T cell expressed and secreted; TNF: tumour necrosis factor; VEGF: vascular endothelial growth factor.
(TIF)

S8 Fig Heatmap showing soluble factors concentrations in the CE3b-group and in the CE4-group at T0 and at T1. Each cytokine value was normalized by subtracting the mean cytokine value calculated for each specific cytokine within each patient-group (CE3b or CE4 groups). Subsequently, this value was divided by the standard deviation calculated for the specific cytokine within each patient-group (CE3b or CE4 groups). Colour codes refer to “red” for the highest expression and “green” for the lowest expression levels. Footnotes: CE: cystic echinococcosis; pt: patient; IL: Interleukin; ra: receptor antagonist; FGF: fibroblast growth factor; G-CSF: granulocyte-colony stimulating factor; GM-CSF: granulocyte-macrophage colony-stimulating factor; IFN: interferon; IP: IFN-γ-induced protein; MCP: monocyte chemoattractant protein; MIP: macrophage inflammatory protein; PDGF: Platelet-derived growth factor; RANTES: regulated on activation, normal T cell expressed and secreted; TNF: tumour necrosis factor; VEGF: vascular endothelial growth factor.
(TIF)
