# Supplementary figures and images for: Accuracy of an experimental whole-blood test for detecting reactivation of echinococcal cysts
Source: PLoS Negl Trop Dis. 2021 Aug 20;15(8):e0009648. doi: 10.1371/journal.pntd.0009648 (PMC8378729; doi:10.1371/journal.pntd.0009648)

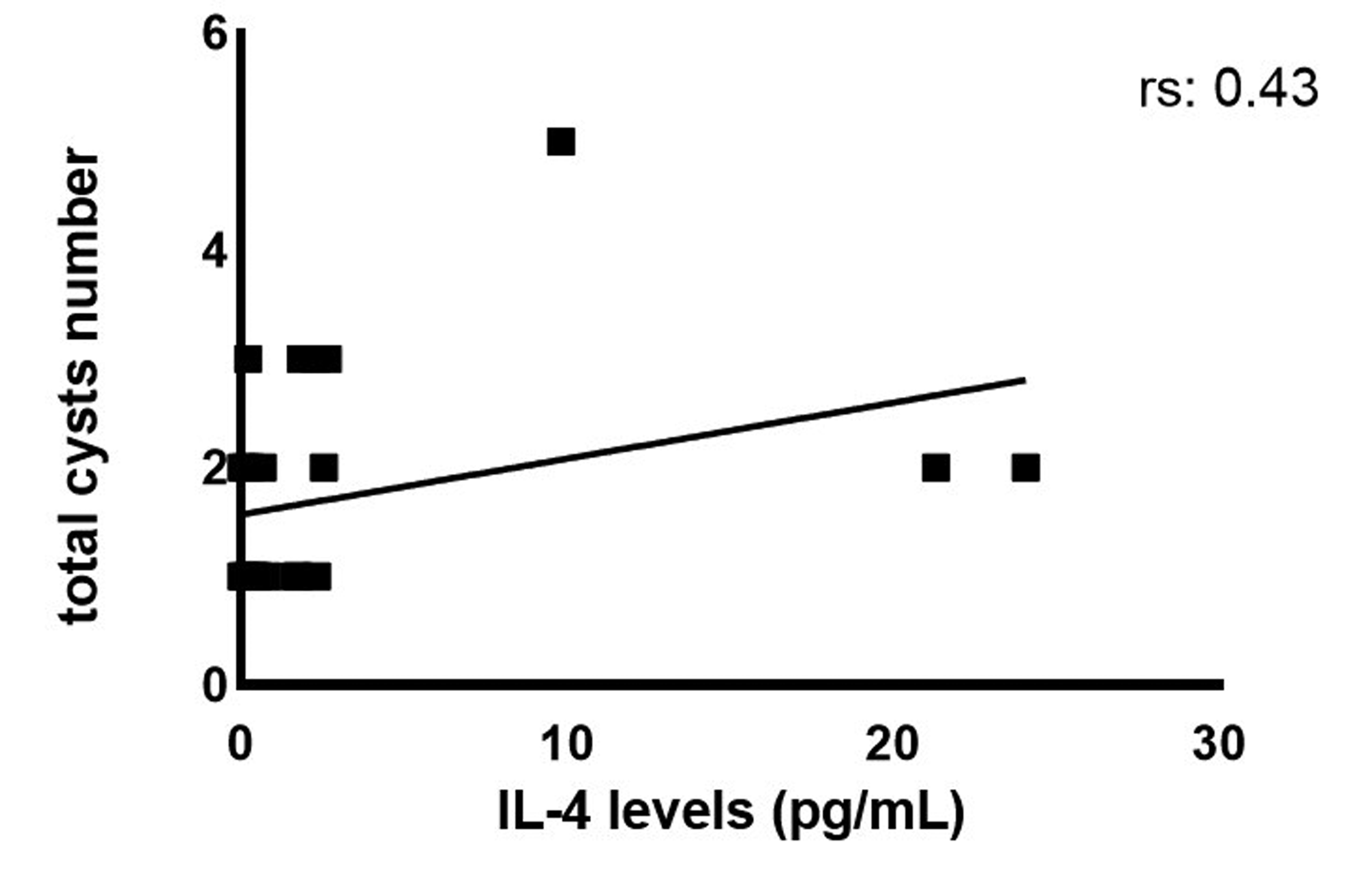

Supplement: S1 Fig — A low but significant positive correlation was found in patients in the CE3b-group between the IL-4 levels and the number of total echinococcal cysts of each patient. Footnotes: IL-4 concentrations were determined by ELISA. Analysis was conducted using Spearman Rank Correlation for correlations (rs>0.7 was considered high correlation, 0.70.5 moderate correlation and rs<0.5 low correlation); differences were considered significant at p-values of ≤0.05. IL: Interleukin. (TIF) [file pntd.0009648.s001.tif]

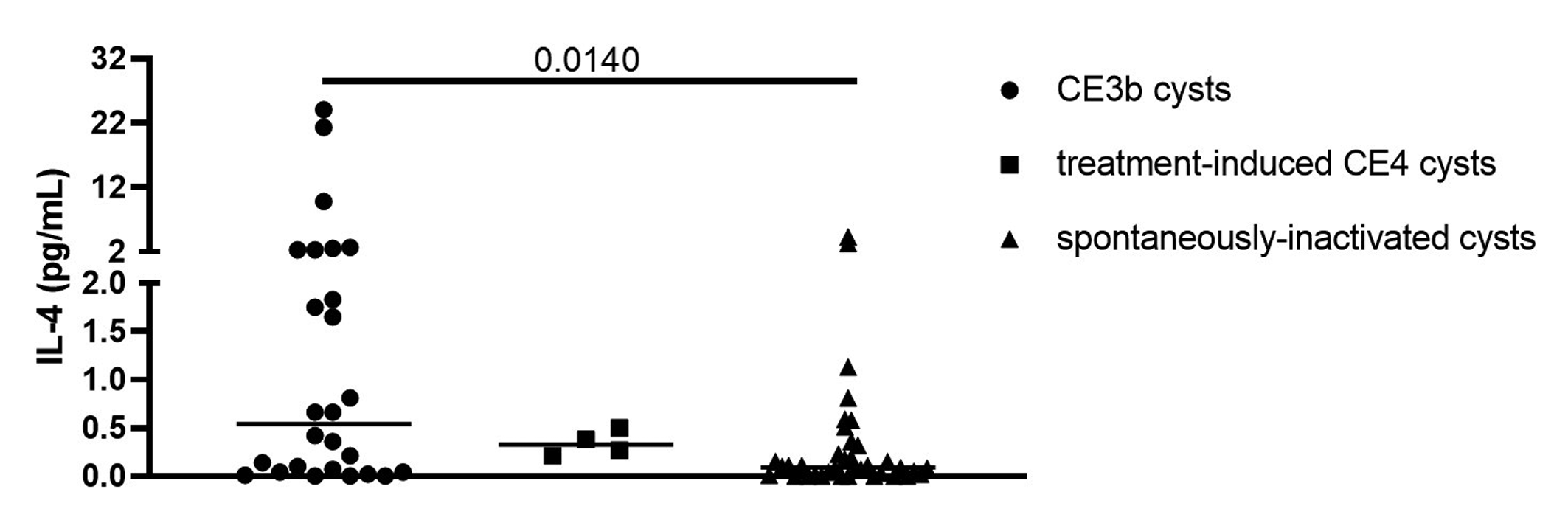

Supplement: S2 Fig — IL-4 levels compared at baseline among patients with CE3b cysts (n = 26 in the CE3b-group), with treatment-induced CE4 cysts (n = 4 in the CE3b-group) and with spontaneously-inactivated cysts (n = 37; CE4-group). The highest IL-4 levels were found in patients with CE3b cysts compared to the other groups. Footnotes: Horizontal bars represent medians. IL-4 concentrations were determined by ELISA. Responses were compared using the Mann-Whitney test with Bonferroni correction; differences were considered significant at p-values of ≤0.016. IL: Interleukin; CE: Cystic Echinococcosis. (TIF) [file pntd.0009648.s002.tif]

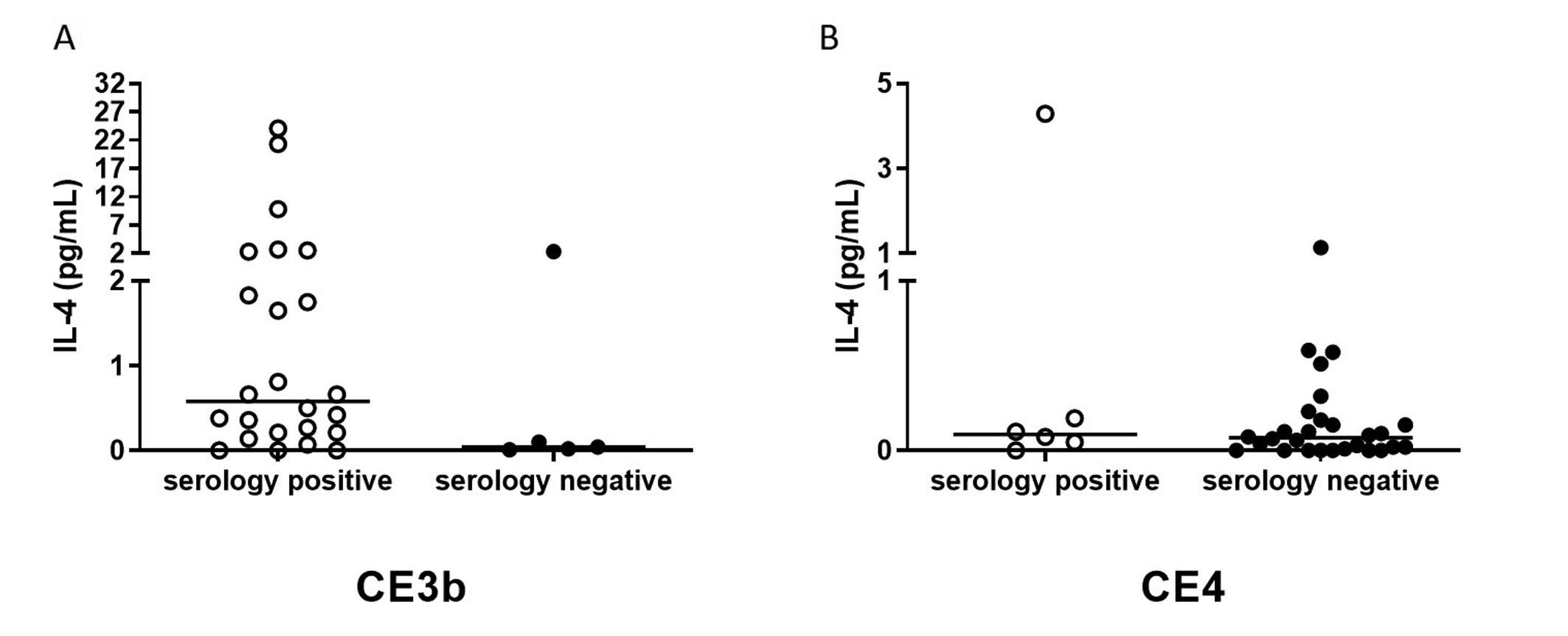

Supplement: S3 Fig — Analysis of IL-4 levels in response to AgB at baseline stratifying the patients based on serology. A. A higher IL-4 response to AgB was observed in patients in the CE3b-group with a positive serology compared to those with negative serology. B. no differences in patients in the CE4-group. Footnotes: Horizontal bars represent medians. IL-4 concentrations were determined by ELISA. Responses in all the panels were compared using the Mann-Whitney; differences were considered significant at p-values of ≤0.05. IL: Interleukin; CE: Cystic Echinococcosis. (TIF) [file pntd.0009648.s003.tif]

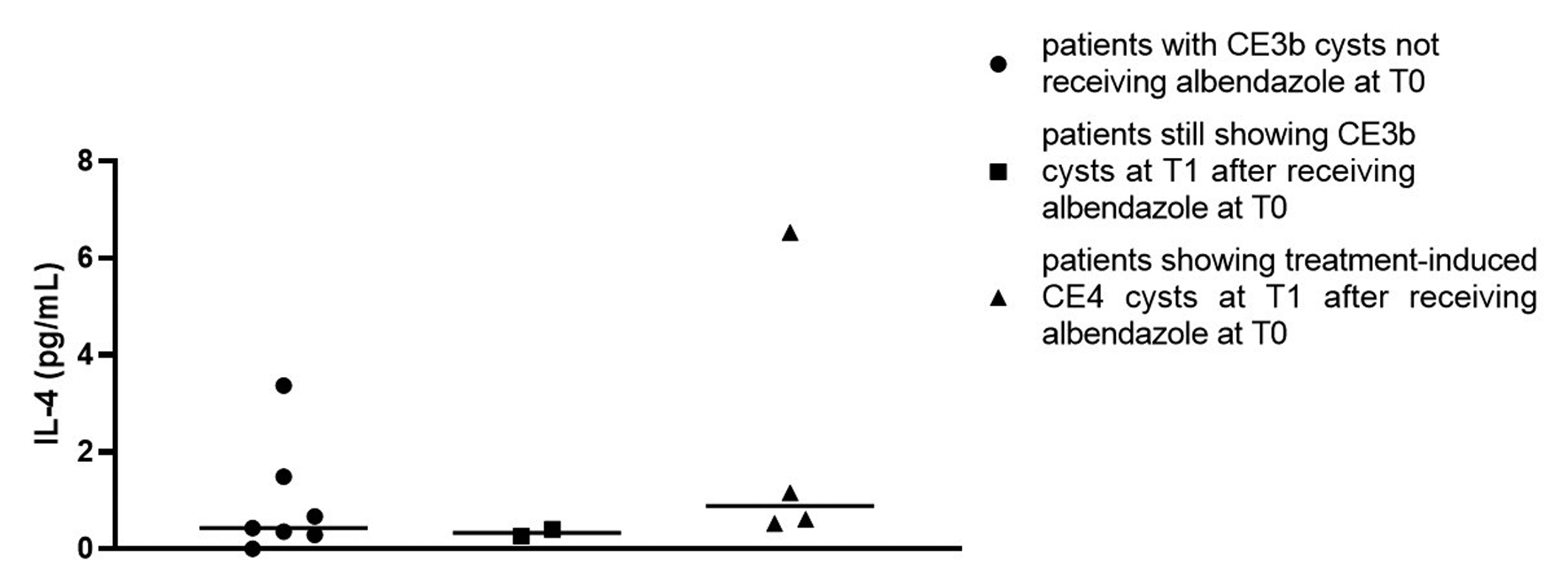

Supplement: S4 Fig — Comparison of IL-4-response comparing patients in the CE3b-group at T1 as follows: patients not receiving albendazole at T0 (n = 7), still showing CE3b cysts at T1 after receiving albendazole at T0 (n = 2), and showing treatment-induced CE4 cysts at T1 after receiving albendazole at T0 (n = 4). The highest IL-4 levels were found in patients with treatment-induced CE4 cysts. Footnotes: Horizontal bars represent medians. IL-4 concentrations were determined by ELISA. Responses were compared using the Mann-Whitney test with Bonferroni correction; differences were considered significant at p-values of ≤0.016. IL: Interleukin; CE: Cystic Echinococcosis. (TIF) [file pntd.0009648.s004.tif]

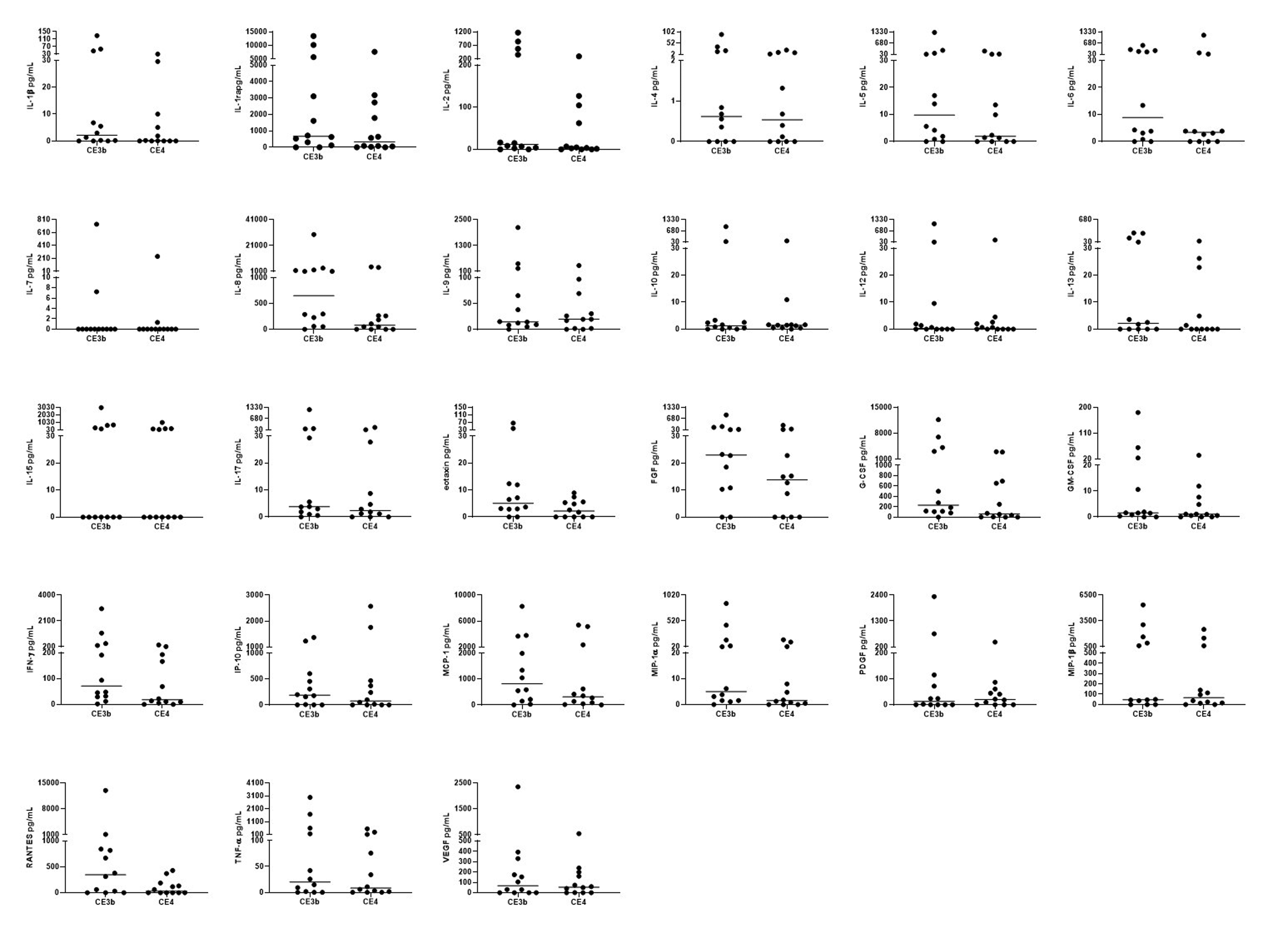

Supplement: S5 Fig — The following soluble factors were evaluated: IL-1β, IL-1ra, IL-2, IL-4, IL-5, IL-6, IL-7, IL-8, IL-9, IL-10, IL-12p70, IL-13, IL-15, IL-17A, Eotaxin, basic FGF, G-CSF, GM-CSF, IFN-γ, IP-10, MCP-1, MIP-1α, MIP-1β, PDGF, RANTES, TNF-α, VEGF. Although patients in the CE3b-group showed higher levels compared to patients in the CE4-group for the majority of the analytes considered, no significant differences were found between the two groups at baseline. Footnotes: Horizontal bars represent medians. Analytes concentrations were determined by luminex. Responses in all the panels were compared using the Mann-Whitney; differences were considered significant at p-values of ≤0.05. IL: Interleukin; ra: receptor antagonist; FGF: fibroblast growth factor; G-CSF: granulocyte-colony stimulating factor; GM-CSF: granulocyte-macrophage colony-stimulating factor; IFN: interferon; IP: IFN-γ-induced protein; MCP: monocyte chemoattractant protein; MIP: macrophage inflammatory protein; PDGF: Platelet-derived growth factor; RANTES: regulated on activation, normal T cell expressed and secreted; TNF: tumour necrosis factor; VEGF: vascular endothelial growth factor. (TIF) [file pntd.0009648.s005.tif]

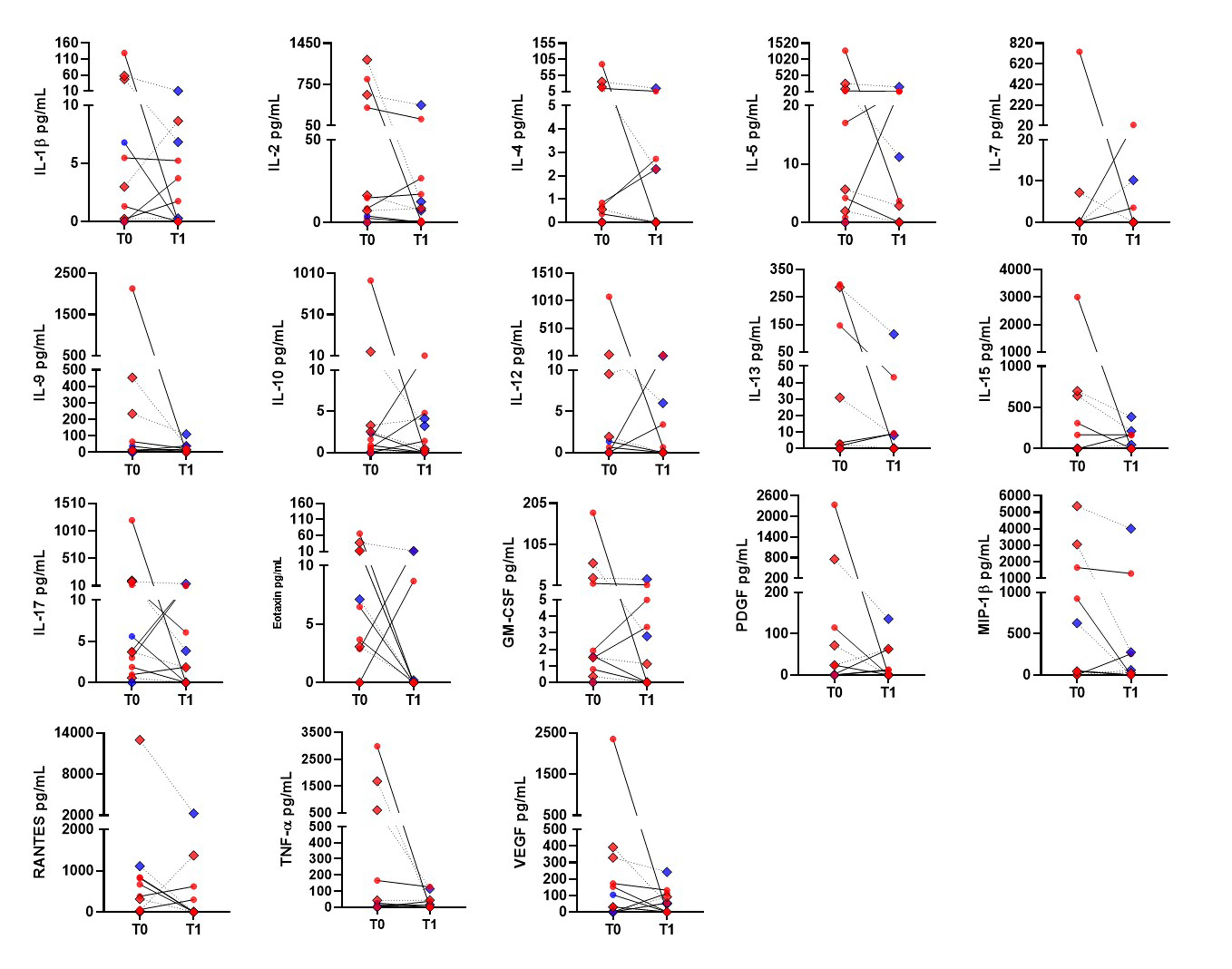

Supplement: S6 Fig — Multiplex analysis performed in 12 patients per study group. The following soluble factors were evaluated: IL-1β, IL-2, IL-4, IL-5, IL-7, IL-9, IL-10, IL-12p70, IL-13, IL-15, IL-17A, Eotaxin, GM-CSF, MIP-1β, PDGF, RANTES, TNF-α, VEGF. None of these factors showed were modulated at T1 compared to baseline. Footnotes: Analytes concentrations were determined by luminex. Responses in all the panels were compared using the Wilcoxon test; differences were considered significant at p-values of ≤0.05. IL: Interleukin; GM-CSF: granulocyte-macrophage colony-stimulating factor; MIP: macrophage inflammatory protein; PDGF: Platelet-derived growth factor; RANTES: regulated on activation, normal T cell expressed and secreted; TNF: tumour necrosis factor; VEGF: vascular endothelial growth factor. (TIF) [file pntd.0009648.s006.tif]

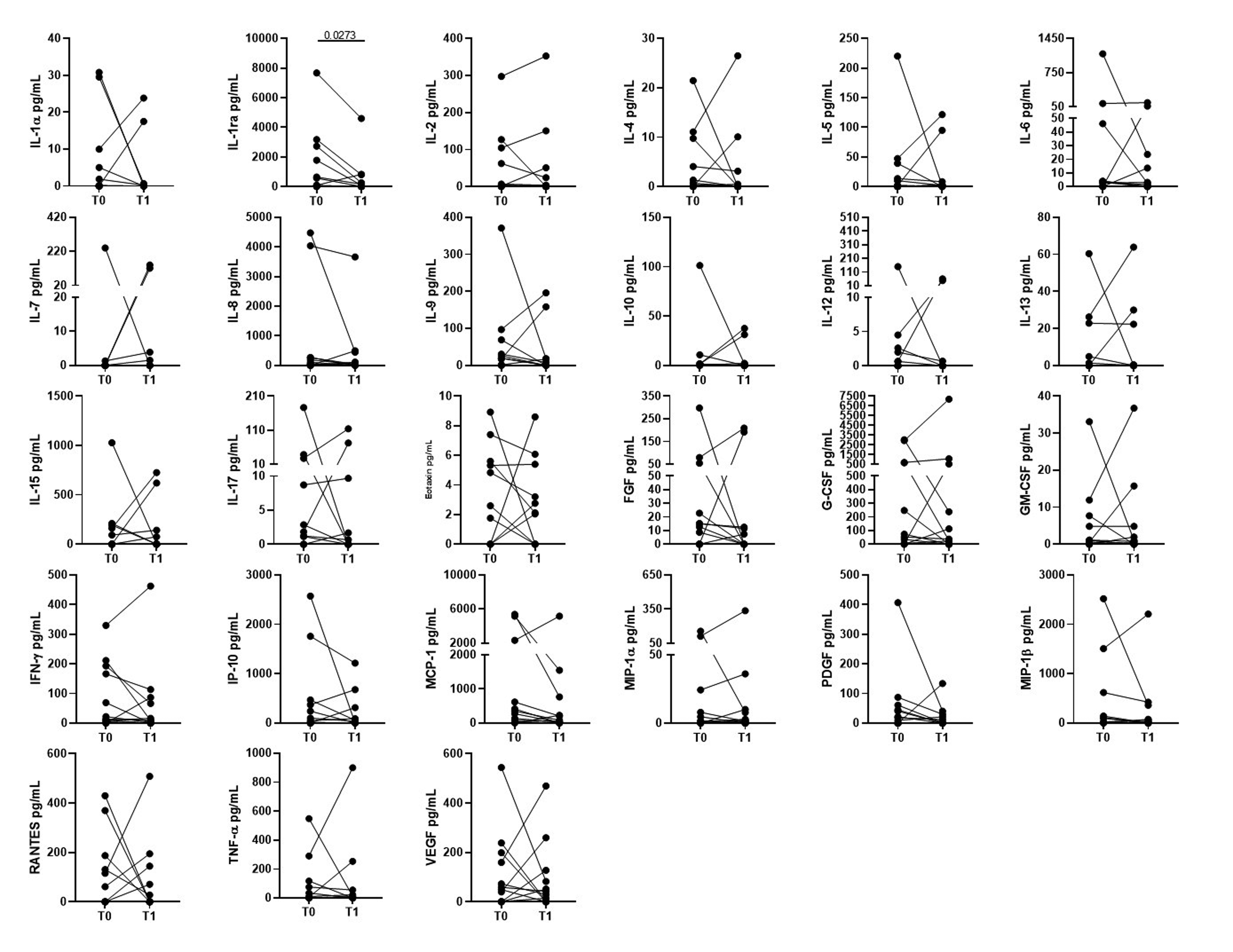

Supplement: S7 Fig — Multiplex analysis performed in 12 patients per study group. The following soluble factors were evaluated: IL-1β, IL-1ra, IL-2, IL-4, IL-5, IL-6, IL-7, IL-8, IL-9, IL-10, IL-12p70, IL-13, IL-15, IL-17A, Eotaxin, basic FGF, G-CSF, GM-CSF, IFN-γ, IP-10, MCP-1, MIP-1α, MIP-1β, PDGF, RANTES, TNF-α, VEGF. No factors, excepted IL-1ra, were modulated at T1 compared to baseline. Footnotes: Analytes concentrations were determined by luminex. Responses in all the panels were compared using the Wilcoxon test; differences were considered significant at p-values of ≤0.05. IL: Interleukin; ra: receptor antagonist; FGF: fibroblast growth factor; G-CSF: granulocyte-colony stimulating factor; GM-CSF: granulocyte-macrophage colony-stimulating factor; IFN: interferon; IP: IFN-induced protein; MCP: monocyte chemoattractant protein; MIP: macrophage inflammatory protein; PDGF: Platelet-derived growth factor; RANTES: regulated on activation, normal T cell expressed and secreted; TNF: tumour necrosis factor; VEGF: vascular endothelial growth factor. (TIF) [file pntd.0009648.s007.tif]

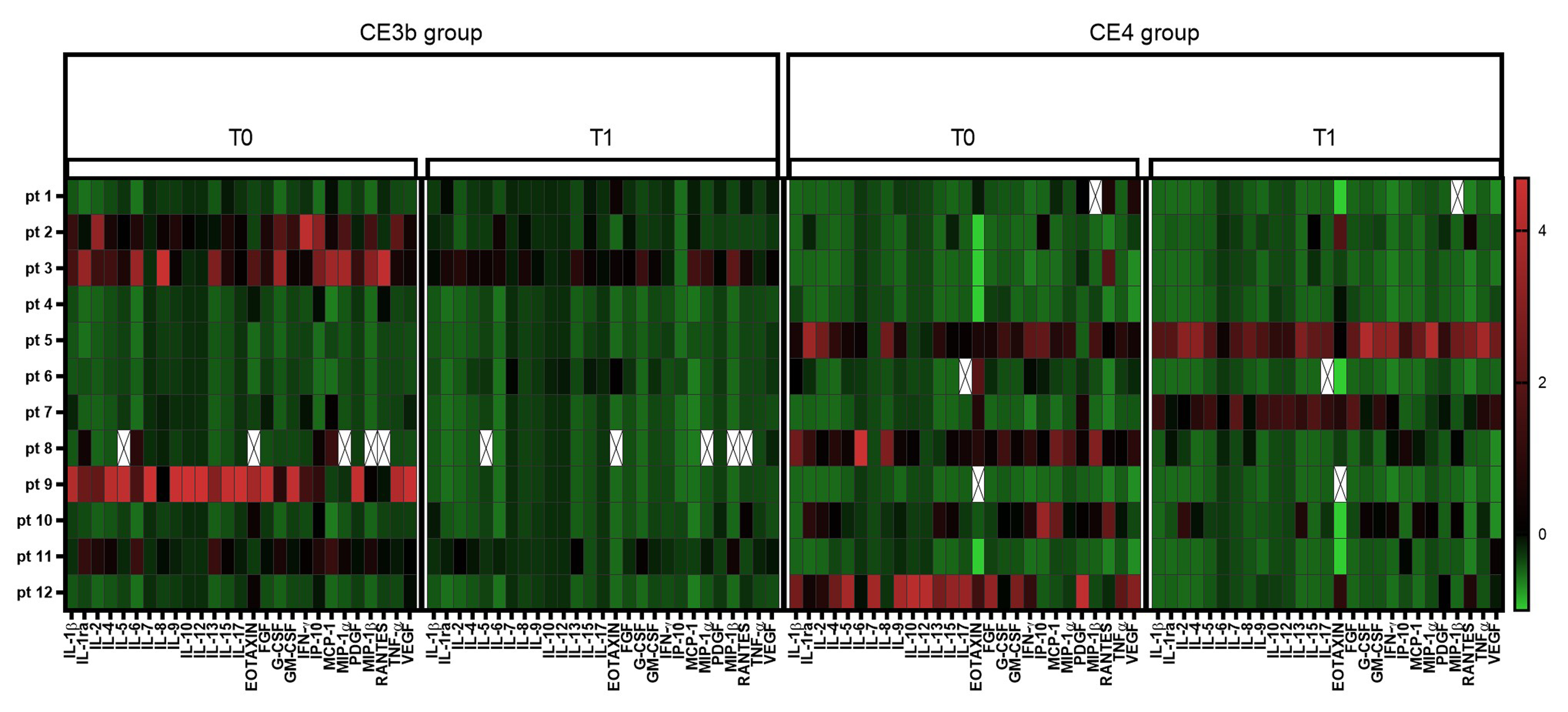

Supplement: S8 Fig — Each cytokine value was normalized by subtracting the mean cytokine value calculated for each specific cytokine within each patient-group (CE3b or CE4 groups). Subsequently, this value was divided by the standard deviation calculated for the specific cytokine within each patient-group (CE3b or CE4 groups). Colour codes refer to “red” for the highest expression and “green” for the lowest expression levels. Footnotes: CE: cystic echinococcosis; pt: patient; IL: Interleukin; ra: receptor antagonist; FGF: fibroblast growth factor; G-CSF: granulocyte-colony stimulating factor; GM-CSF: granulocyte-macrophage colony-stimulating factor; IFN: interferon; IP: IFN-γ-induced protein; MCP: monocyte chemoattractant protein; MIP: macrophage inflammatory protein; PDGF: Platelet-derived growth factor; RANTES: regulated on activation, normal T cell expressed and secreted; TNF: tumour necrosis factor; VEGF: vascular endothelial growth factor. (TIF) [file pntd.0009648.s008.tif]
